# The Chemical Residues in Secondary Beekeeping Products of Environmental Origin

**DOI:** 10.3390/molecules29163968

**Published:** 2024-08-22

**Authors:** Joanna Wojtacka

**Affiliations:** Department of Veterinary Public Health, Faculty of Veterinary Medicine, University of Warmia and Mazury in Olsztyn, 10-718 Olsztyn, Poland; joanna.wojtacka@uwm.edu.pl; Tel.: +48-89-523-33-31

**Keywords:** secondary beekeeping products, residues, propolis, bee pollen, bee bread, environmental hazards, maximum residue limits, public health, regulations

## Abstract

Natural products of bee origin, despite their complex composition and difficulties in standardization, have been of high interest among scientists representing various disciplines from basic sciences to industrial and practical implementation. As long as their use is monitored and they do not impact human health, they can be considered valuable sources of many chemical compounds and are potentially useful in medicine, food processing, nutrition, etc. However, apart from honey, the general turnover of bee products lacks precise and detailed legal requirements ensuring their quality. The different residues in these products constitute a problem, which has been reported in numerous studies. All products derived from beekeeping are made by bees, but they are also influenced by the environment. Such a dual pathway requires detailed surveillance of hazards stemming from outside and inside the apiary. This should be ensured via harmonized requirements arising from the binding legal acts, especially in international and intercontinental trade zones.

## 1. Introduction

There are two major divisions of traditional bee products that provide a better understanding of the way they are produced and subject to spoilage or contamination. Based on the economic aspect of beekeeping, bee products can be divided into primary (honey and wax) and secondary (bee pollen, bee bread, propolis, royal jelly and bee venom) classes.

Another division concerns the way bee products are produced. They can be manufactured by bees, like wax, royal jelly, and venom, or processed by bees like honey, bee pollen, bee bread, and propolis. The latter group requires both environmental resources and the addition of substances of bee origin, making it the most prone to contamination from different residues coming from outside and inside the apiary and related to human activity. So far, it has not been analyzed how bee products are affected by the interactions of pesticides of environmental origin and Veterinary Medicinal Products (VMPs) used in beekeeping. There are studies wherein they were screened, but the results were presented without attempting to analyze if and how they interact [1,2]. However, the impact of agropesticides and VMPs on individual bees and bee colonies is widely studied and regularly reviewed [3,4,5,6]. Clearly, the presence of residues of the compounds of both of these groups should eliminate bee products from the food chain, as the pesticide residue content of hive products is a food safety concern [7,8]. They should also be excluded from use in the pharmaceutical industry. However, few authors focus on this problem, and it is mainly the bioactive, health-promoting, and nutritional characteristics of secondary beekeeping products that are highlighted [9,10,11]. At the same time, the ordinary consumer assumes in advance that the safety of food or medicinal products is guaranteed by appropriate services using previously developed law regulations and standards including validated chemical analyses and established maximum residue levels (MRLs), with the latter indicating that the upper legal level of a concentration of a pesticide residue in or on food or feed is at the lowest exposure level necessary to protect the consumer [12]. Thus, based on EU law, it is also possible to divide beekeeping products into honey and four other bee products, i.e., wax, royal jelly, propolis, and bee pollen, and only for propolis does a direct distinction between edible and inedible exist [13]. No regulations at the EU level directly mention bee bread, which can be categorized under “other apiculture products” [12]. The term “apiculture products” encompasses honey, beeswax, royal jelly propolis, and pollen [13]. In the model of the official certificate for entry into the EU [14] secondary beekeeping products are described as other apiculture products intended for human consumption and are treated the same way as honey according to the so-called HON model. Such an approach and nomenclature potentially allow the turnover of edible secondary beekeeping products other than propolis. The cited document consists of two parts, i.e., a Description of Consignment and a Certification that attests to public health safety according to the core legal acts of food law assuring standardized quality, safety, and no harmful effects on human health and life [15,16,17,18,19], meaning these products must be free from any residues detected at their corresponding MRLs.

Edible bee products that are subjected to verification using these means of health attestation can be classified under eight different digital HS (the shorts forms for Harmonized Commodity Description and Coding System) codes, e.g., natural honey (0409), bees wax (1521), other sugars, and artificial honey mixed with natural honey (1702). Five of these codes let edible secondary beekeeping products such as bee pollen, bee bread, and propolis exist in legal trade and turnover and are classified as follows: edible products of animal origin, not elsewhere specified or included (0410), other animal products used in the preparation of pharmaceutical products that are fresh, chilled, frozen, or otherwise provisionally preserved (0510), other vegetable products (1212) and food preparations not elsewhere specified or included (2106) [14]. Moreover, all the turnover data on edible secondary beekeeping products are registered in the Information Management System for Official Controls (IMSOC), which is run and supervised by the European Commission.

Bee products, including secondary beekeeping products, are an important source of bioactive compounds that have relevant biological effects and engage in numerous biological activities. They have antimicrobial, anti-inflammatory, antioxidant, anticancer, immune-modulatory, antidiabetic, hypolipidemic, hypotensive, and anti-allergic properties [20,21,22,23,24,25,26]. Nevertheless, such multidirectional characteristics are insufficient for secondary beekeeping products to be considered acceptable in the healthcare system due to the lack of standardization [11]. Moreover, the lack of uniform legal regulations for particular secondary beekeeping products, such as the regulation that exists for honey [19], complicates any kind of standardization. For example, these products have been proven to be affected by chemical hazards, and this is the case not only for bee products stemming from *Apis mellifera* but also other members of Apidae [27]. This aspect has relevance regarding food safety and quality; customs in international turnover, especially in terms of import from countries without EU-approved residue control; proper labeling; the provisions of reliable information to consumers; and public notifications on food fraud. The above is reflected in a few notifications regarding bee products other than honey and royal jelly, with thousands of notifications being available in the Rapid Alert System for Food and Feed (RASFF), which is run by the European Commission and open to the public [28]. 

The aim of this review was to indicate the importance of standardizing the methodologies used in pesticide detection in bee secondary products and supplement the legal system with provisions regulating their statuses.

## 2. Secondary Beekeeping Products

### 2.1. Bee Pollen 

Bee pollen can be described as natural flower pollen collected from plants by bees. The bees bring it to the hive in the form of pollen grains stuck together in lumps [29] thanks to the secretion of their salivary glands and nectar. The bees transport pollen in the baskets on their hind legs to the hive. A colony of bees can collect between 50 and 250 g of pollen per day, amounting to 15–40 kg yearly. The main function of pollen is to serve as a source of nutrients for the colony, thus guaranteeing its development and maintenance [11]. Bee pollen can be unifloral or multifloral depending on the source of forage, which also determines the resulting pollen’s chemical profile and further use [30]. Principally, there are two forms of bee pollen available on the market. These products can be sold fresh-frozen or traditionally dried, although lyophilized products are also available, and lyophilization has been proven to preserve the biochemical activity and content of bee pollen [31,32]. The processing method and the conditions of storage can significantly impact these products’ quality and biochemical activity [31,32,33,34]. There are some macroscopic and organoleptic features that may disqualify bee pollen from being used in these products. These include the presence of all forms of pollen pests such as beetles, butterflies, and mites. Moreover, the presence of mold, dead bees and their parts, plant parts, soil or sand, and a foreign smell or taste is unacceptable [29]. 

Bee pollen’s chemical composition and nutritional values vary depending on its geographical and botanical origin. This phenomenon is naturally regulated and it has been studied and analyzed for years [35,36,37]. Human activity, anthropopressure in apiculture and agriculture, and the procedures used in food production also impact the chemical composition of this secondary beekeeping product [38,39]. The contamination of bee pollen with pesticides can reach over 60% [40]. A detailed review of the contamination of bee pollen with pesticides stemming from veterinary interventions in bee colonies, crop management, and natural sources has been presented elsewhere [8,41]. It is worth mentioning that the hazards have been confirmed not only in bee pollen collected in apiaries but also in samples taken from products available on the market, i.e., from herbal, grocery, and online stores [42]. Therefore, some authors have indicated that there is a lack of regulations concerning the supervision of the hazards affecting bee pollen. RASFF statistics, as given in Table 1, show notably low numbers of notifications regarding chemical hazards in this secondary beekeeping product.

### 2.2. Bee Bread

Bee bread is one of the least-investigated secondary beekeeping products [11], despite the fact that it is essential for the proper development of a bee colony. It is irreplaceable feed for older worker bee larvae and young bees producing royal jelly. It consists of bee pollen that is laid in the cells of the comb, mixed with the secretion of the bees’ salivary glands and honey, and tamped tightly to protect it from air exposure [10]. In anaerobic conditions, it undergoes lactic acid fermentation, which further preserves it and makes it an easily digestible product. It has a similar composition to bee pollen but with remarkable quantitative differences, mainly due to its fermentation process [9]. Depending on the intensity of pollen flow and the methods employed for collecting corbicular pollen, it is possible to conduct bee bread harvesting and subsequently achieve a positive economic outcome [43], mainly due to its considerable pharmacological properties, which have been verified in vitro and in vivo and offer a wide field for the exploitation of its benefits in the food and pharmaceutical industries [10]. Although none of the EU regulations directly consider bee bread either as an edible or inedible product, it can be assumed that its quality and safety can be considered partially regulated because it can undoubtedly be enumerated within the group defined as other apiculture products. The way in which bee bread is produced reveals that the paths of probable contamination and residue presence in bee bread involve overlapping hazards related to honey and bee pollen and therefore are also related to liphophilic and hydrophilic components. A recent study conducted in Switzerland showed that a biweekly sampling procedure can allow the capture of the peaks of pesticide residues in bee bread [44]. In this study, contamination levels similar to bee pollen [40] could even reach up to 60% of the samples tested, with significant differences between the bee colonies from the same location. Due to its complex composition and biological properties, bee bread, if free from chemical hazards, can be used as a food supplement at the daily dosage of about 20–40 g for an adult for regeneration and strengthening with no risk of overdosage [45]. When compared to pollen, bee bread, due to its greater biological value, faster digestibility, and superior chemical composition, is perceived as a much more valuable secondary beekeeping product [46].

### 2.3. Propolis

Bees produce propolis using plant resins. Depending on their availability in the environment there is also a variable content of wax added, which has proved to be a significant medium for pesticide residues in propolis [47]. Terpenes and aromatic oils are enumerated as other natural constituents. There are some macroscopic and organoleptic features that may disqualify bee propolis from commercialization. These include the presence of wax flukes and other worms at all stages of development, asphalt particles, window putty, mold, or a foreign smell. Propolis should have a yellow to dark brown color, often with a greenish and reddish tinge; a pleasant, balsamic smell; and a brittle or plastic consistency [48]. A bee colony uses propolis to seal the hive and preserve any biological material that can become hazardous due to spoilage. The properties of propolis, similar to other secondary beekeeping products, depend on its biological and geographical origin. Different types of propolis are classified based on phenolic-resinous fraction studies [49]. The general standardization of propolis characteristics can thus be difficult; however, some types of propolis, i.e., Gerês propolis, exhibit surprisingly consistent ranges of analyzed parameters that can facilitate the future validation of reference parameters [50]. However, few, very general records regarding propolis in the regulations make it a difficult product to validate in terms of the assurance of public health. This is due to multi-contamination and a cumulative effect that applies to residues of both authorized and no-longer-authorized, e.g., bromopropylate, pesticides. There are results showing that over 60% of propolis samples analyzed were not suitable for human consumption due to contamination with pesticides [51]. The chemical hazards in propolis and propolis-based products have not been detected in some of the samples analyzed [52] but they have been revealed to be present in a few stages of the food chain [53]. When ingested at high concentrations (>3000 μg/kg) these hazards may pose acute risk in humans [7]. A review of the contamination of propolis with pesticides used in veterinary interventions in bee colonies has been presented elsewhere [54]. 

The reports available in RASFF and prepared based on official control measures aimed at verifying the safety and quality of propolis and propolis-based products present only three incidents regarding this secondary beekeeping product (Table 2).

## 3. Pesticide Residues

The presence of chemical residues in secondary beekeeping products poses a risk to human health and affects honey bees, including bees living far from agricultural regions [27,55]. In the foraging season, pesticides impact bee lifespan and the survival of bee colonies in the winter. They also decrease bees’ foraging activity and pollination services by acting as neuroinhibitors by impeding the acetylcholine neurotransmitter action in the insect nervous system [56] and thus influencing olfactory sensation, memory, ability to navigate back to the nest, flight ability, and dance circuits [57]. The activity of pesticides contaminating bee products is complex, and often the metabolites show more negative effects than the parent compound. Toxicokinetics and bioaccumulation are not the only features that should be considered when assessing the hazards posed by pesticides. When ingested, pesticides enter the human body alongside bee products directly. The consumption of secondary beekeeping products by humans on a long-term basis is unlikely; however, it can undoubtedly result in chronic clinical and neurobehavioral disorders [58]. Thus, the principal objective of the food laws in the EU, that is, to ensure a high level of consumer protection with regard to food safety [17], cannot be achieved when bee products are contaminated. This is why the detection of chemical residues is necessary, despite the fact that bee products are complex matrices. Chemical analyses start with sample preparation with the use of different methods and continue with detection based on high-performance liquid chromatography with tandem mass spectrometry (HPLC-MS/MS) [59,60,61,62]. For some pesticides, a less expensive LC–diode array detector (DAD) combined with liquid chromatography can also be used for routine monitoring [63]. Pesticide residues of environmental origin in secondary beekeeping products appear with and without human participation [54,64]. Both ways may result in a toxic effect in humans when a bee product enters the food chain in concentrations exceeding MRLs, often as a multiresidue matrix or as a result of accumulation and exceeding daily acceptable intake (ADI) [7,40,65,66]. Although food processing by worker bees contributes to the degradation and metabolization of pesticides in the hive [67], according to EU law [12], there are a number of pesticide residues whose maximum levels are established for food and feed of plant and animal origin in order to ensure a high level of consumer protection. These limits and requirements are established based on good agricultural practice (GAP), which means the nationally recommended, authorized or registered safe use of plant protection products under actual conditions at any stage of the food chain. The pesticide residues in question include active substances, metabolites, and/or the breakdown or reaction products of active substances currently or formerly used in plant protection products or as biocides in veterinary practice when applied directly to the hive in different pharmaceutical forms, as shown in Table 3. 

European regulations directly indicate that MRLs should be set for pharmacologically active substances used or intended to be used in VMPs placed on the market in the community [69]. However, MRLs of pharmacologically active substances from VMPs in secondary beekeeping products are applied for coumafos, fluvalinate and tau-fluvalinate [12]; for other active substances, e.g., amitraz, MRLs established for honey are used [70]. Among the products of plant and animal origin to which MRLs are applied are honey and other apicultural products (Code number 1040000), with the proviso that the products subject to this requirement are identified and listed within this group. The MRLs established for secondary beekeeping products are given for individual pesticides or a pesticide and its isomers, salts, corresponding compounds, esters, conjugates or reaction products. In total, there are 76 different compounds plus the group of pyrrolizidine alkaloids specified for pollen, 1 plus the sum of three polycyclic aromatic hydrocarbons (PAHs) for propolis, and 186 more for pollen and propolis, and products specified in the definition as apiculture products and the default for bee bread. All pesticides of environmental origin with legally established MRLs in the EU) are given in Table 4.

## 4. Conclusions

The hazards posed by pesticide residues present in secondary beekeeping products are generally divided into two numerically unequal groups: one numerous group comprising mainly residues originating from human activity in agriculture and apiculture reported in scientific publications, and a second group that is numerically irrelevant and involves single cases of the presence of pesticides of natural origin reported in a few official documents. Such a discrepancy indicates that the regulatory system for the protection of public health is inefficient in terms of the safety and quality of secondary beekeeping products like bee pollen, bee bread and propolis. National programs devoted to biomonitoring with the use of bee products are valuable sources of information but cannot be perceived as a global solution to the problem of the contamination of bee products with pesticides. Their standardization is possible only when the supervising authorities are equipped with the legal force required to ensure regular testing. Discussion over the beneficial effects of bee products on human health and the possibility of using them as alternatives to traditional medicinal products is warranted only in cases where these products are free from hazardous components. 

## Figures and Tables

**Table 1:** Pesticides revealed in pollen as a result of official controls in EU countries or at the borders of the EU according to RASFF [28].

Category of Matrix	Origin/ Notifying Country	Type of Hazard Analytical Result/Max.	Type of Notification	Measures Taken
Other food product/ mixed	France/Switzerland	Pyrrolizidine alkaloids in pollen 3300 µg/kg/500 µg/kg	Information notification for follow-up	Recall from consumer
Other food product/ mixed	Spain/ Belgium	Pyrrolizidine alkaloids in pollen 1430 µg/kg/500 µg/kg	Alert notification	Withdrawal from the market Informing recipients Recall from consumer
Other food product/mixed	Spain/ Belgium	Pyrrolizidine alkaloids in pollen 1070 µg/kg/500 µg/kg	Alert notification	Informing recipients Recall from consumer
Herbs and spices	Albania/Croatia	Metobromuron in Chamomille pollen No data	Border rejection notification	Re-dispatch
Other food product/mixed	Poland/ Poland	Pyrrolizidine alkaloids in pollen 1187 ± 301 µg/kg/500 µg/kg	Alert notification	Public warning—press release
Feed materials	Spain/Netherlands	Matrine, Amitraz and Pyrrolizidine alkaloids in organic bee feed Matrine 0.097 mg/kg/0.010 mg/kg Amitraz 0.042 mg/kg/0.010 mg/kg Pyrrolizidine alkaloids 880 µg/kg/500 µg/kg	Information notification for follow-up	Informing consignor Detained by operator

**Table 2:** Pesticides and other types of hazards revealed in propolis as a result of official control measures in EU countries or at the borders of the EU according to RASFF [28].

Category of Matrix	Origin/ Notifying Country	Type of Hazard Analytical Result/Max.	Type of Notification	Measures Taken
Honey and royal jelly	China/ Germany	Chloramphenicol in propolis extract 0.96 µg/kg/na ^1^	Information Notification for attention	Withdrawal from the market
Dietetic foods, food supplements and fortified foods	UK/ Portugal	Composite products (Gelatine capsules) Hazard not defined	Border rejection notification	Product (to be) redispatched or destroyed
Dietetic foods, food supplements and fortified foods	Turkey/Germany	Missing warning notice for Propolis throat spray with honey Labelling—insufficient— labelling absent/ incomplete/incorrect	Information Notification for follow-up	Detained by operator Removal of online offer Informing recipient(s)

^1^ prohibited substance.

**Table 3:** VMPs authorized for use on honey bees in the EU/EEA [68].

Pesticide Used as VMPs	Active Substance	Possible Drug Form
Synthetic acaricides		
Pyrethroids	Tau-fluvalinate	Beehive strips, beehive solution, concentrate for nebulizer solution
	Fluvalinate	
	Flumethrine	
Organophosphorous compounds	Coumaphos	Tablets, concentrate for cutaneous solution, beehive strips, concentrate for dip emulsion
Formamidines	Amitraz	Solution for beehive strip, beehive strip, bee-smoke stick, cutaneous solution, beehive solution
Complex VMPs	Tau-fluvalinate/Amitraz	Solution for beehive strip,
Natural acaricides		
Organic acids	Sipelghape (formic acid)	Nebuliser suspension, in-hive use solution, cutaneous solution, beehive-strip, nebulization solution, oral solution, beehive solution, powder for oral solution, powder and solution for beehive dispersion, cutaneous spray,
	Sipelgaphe iodine	
	Oxalic acid	
	Oxalic acid dihydrate	
	Oxalic acid/Sipelghape	
Thymol and thymol-based	Thymol	Beehive gel, beehive strip, inhalation vapour tablet
	Cineole	
	Camphor racemic	
	Levomenthol	
	Eucalyptus oil	
	Natural menthol	
	Peppermint	
	D-camphor (1*R*, 2*S*, 5*R*)-5-methyl-2-(1-methylethyl)-cyclohexanol	

**Table 4:** Residues of pesticides of environmental origin specified in the EU regulations and their MRLs in secondary beekeeping products [12,71].

Legal Definition of Secondary Beekeeping Products	Pesticide Residue/s	MRLs
Pollen	Dimethomorph (sum of isomers), Clodinafop and its isomers and their salts, expressed as clodinafop, Clomazone, Metconazole (sum of isomers), Prosulfocarb, Beflubutamid, Fenpropidin (sum of fenpropidin and its salts, expressed as fenpropidin), Formetanate (sum of formetanate and its salts expressed as formetanate hydrochloride), Tebuconazole, Fludioxonil, Chlorotoluron, Trinexapac (sum of trinexapac (acid) and its salts, expressed as trinexapac, Propamocarb (sum of propamocarb and its salts, expressed as propamocarb), Benthiavalicarb [Benthiavalicarb-isopropyl (KIF230 R-L) and its enantiomer (KIF-230 SD) and its diastereomers (KIF-230 S-L and KIF230 R-D), expressed as benthiavalicarb-isopropyl], Forchlorfenuron, Nicosulfuron, Dicloran, Dinocap (sum of dinocap isomers and their corresponding phenols expressed as dinocap), Benfluralin, Cyprodinil, Metaldehyde, Bifenox, Diflufenican, Flutolanil, Spinosad (spinosad, sum of spinosyn A and spinosyn D), Isopyrazam, Imazapic, Isoprothiolane, Saflufenacil (sum of saflufenacil, M800H11 and M800H35, expressed as saflufenacil), Imazapyr, Dichlobenil, Tolylfluanid (sum of tolylfluanid and dimethylaminosulfotoluidide expressed as tolylfluanid), Trifluralin, Vinclozolin, Cyclanilide, Diniconazole (sum of isomers), Butralin, Quinoclamine, Flusilazole, Procymidone, Propisochlor, Propanil, 1,3-Dichloropropene, 2-naphthyloxyacetic acid, Acetochlor, Chloropicrin, Flurprimidole, 2,4,5-T (sum of 2,4,5-T, its salts and esters, expressed as 2,4,5-T), Barban, Bromophos-ethyl, Camphechlor (Toxaphene), Chlorbufam, Chloroxuron, Chlozolinate, Di-allate (sum of isomers), Dinoseb (sum of dinoseb, its salts, dinoseb-acetate and binapacryl, expressed as dinoseb), Dinoterb (sum of dinoterb, its salts and esters, expressed as dinoterb), Dioxathion (sum of isomers), DNOC, Ethylene oxide (sum of ethylene oxide and 2-chloro-ethanol expressed as ethylene oxide), Fentin (fentin including its salts, expressed as triphenyltin cation), Flucycloxuron, Flucythrinate [flucythrinate including other mixtures of constituent isomers (sum of isomers)], Formothion, Mecarbam, Methacrifos, Monolinuron, Propham, Pyrazophos, Quinalphos, Resmethrin [resmethrin including other mixtures of consituent isomers (sum of isomers)], Tecnazene	0.05 ^1 ^ mg/kg
Pollen	Metazachlor (sum of metabolites 479M04, 479M08 and 479M16, expressed as metazachlor)	0.08 mg/kg
Pollen	Anthraquinone	0.02 ^1^ mg/kg
Pollen	Didecyldimethylammonium chloride (mixture of alkyl-quaternary ammonium salts with alkyl chain lengths of C8, C10 and C12)	to be reviewed by 22 February 2030
Pollen	Benzalkonium chloride (mixture of alkylbenzyldimethylammonium chlorides with alkyl chain lengths of C8, C10, C12, C14, C16 and C18)	to be reviewed by 22 February 2030
Pollen (including pollen based food supplements and pollen products)	Pyrrolizidine alkaloids the maximum level: - refers to the lower bound sum of 21 pyrrolizidine alkaloids - applies to the food supplements as placed on the market	500 μg/kg
Propolis	Benzo(a) pyrene	10.0 μg/kg
Propolis	Sum of PAHs: benzo(a) pyrene, benz(a) anthracene, benzo(b) fluoranthene and chrysene	50.0 μg/kg
Other apiculture products	Dimoxystrobin, Metrafenone, Amidosulfuron, Halauxifen-methyl [sum of halauxifen-methyl and X11393729 (halauxifen), expressed as halauxifenmethyl], Flumetralin, Mandestrobin, Dichlorprop [sum of dichlorprop (including dichlorpropP), its salts, esters and conjugates, expressed as dichlorprop], Haloxyfop [sum of haloxyfop, its esters, salts and conjugates expressed as haloxyfop (sum of the R- and S-isomers at any ratio)], Diethofencarb, Flonicamid (sum of flonicamid, TFNA and TFNG expressed as flonicamid), Flutriafol, Pirimicarb, Prothioconazole: prothioconazole-desthio (sum of isomers), Teflubenzuron, Clothianidin, Thiamethoxam, Tolclofosmethyl, Benzovindiflupyr, Dodine, Glufosinate (sum of glufosinate isomers, its salts and its metabolites 3-[hydroxy(methyl)phosphinoyl] propionic acid (MPP) and N-acetylglufosinate (NAG), expressed as glufosinate), Fluazifop-P (sum of all the constituent isomers of fluazifop, its esters and its conjugates, expressed as fluazifop), Fuberidazole, Fluoxastrobin (sum of fluoxastrobin and its Z-isomer), Cyantraniliprole, Isofetamid, Sulfoxaflor (sum of isomers), Cymoxanil, Phosphane and phosphide salts [sum of phosphane and phosphane generators (relevant phosphide salts), determined and expressed as phosphane], Aclonifen, Fluazinam, Sulcotrione, Triflusulfuron (6-(2,2,2-trifluoroethoxy)-1,3,5-triazine-2,4-diamine (INM7222), Oxathiapiprolin, Fenpyroximate, Triadimenol (any ratio of constituent isomers), Tebufenpyrad, Acrinathrin, Propargite, Bensulfuron-methyl, Dimethachlor, Lufenuron (any ratio of constituent isomers), Triclopyr, Fenpicoxamid, Penoxsulam, Etofenprox, Paclobutrazol (sum of constituent isomers), Bromuconazole (sum of diastereoisomers), Fenpyrazamine, Pyridaben, Mefentrifluconazole, Flutianil, Florpyrauxifen-benzyl, Fenoxycarb, Quizalofop [sum of quizalofop, its salts, its esters (including propaquizafop) and its conjugates, expressed as quizalofop (any ratio of constituent isomers)], Tebufenozide, Mandipropamid (any ratio of constituent isomers), Profoxydim, Cyflufenamid [sum of cyflufenamid (Z-isomer) and its E-isomer, expressed as cyflufenamid], Fenbuconazole (sum of constituent enantiomers), Fluquinconazole, Tembotrione [sum of parent tembotrione (AE 0172747) and its metabolite M5 (4,6-dihydroxy tembotrione), expressed as tembotrione], Napropamide (sum of isomers), Sintofen, Chromafenozide, Fluometuron, Pencycuron (sum of pencycuron and pencycuronPB-amine, expressed as pencycuron), Sedaxane (sum of isomers), Fluvalinate (sum of isomers) resulting from the use of tau-fluvalinate, Bupirimate, Ethirimol, Pyriofenone, Flufenoxuron, Phosalone, Fluopicolide, Proquinazid, Pyridalyl, Sum of diclofopmethyl, diclofop acid and its salts, expressed as diclofop-methyl (sum of isomers), Fluopyram, Ipconazole, Terbuthylazine, Fluxapyroxad, Hymexazol, Metamitron, Penflufen (sum of isomers), Difluoroacetic acid (DFA), Flupyradifurone, Bixafen, Fenazaquin, Spinetoram (sum of spinetoram-J and spinetoram-L), Tefluthrin [tefluthrin including other mixtures of constituent isomers (sum of isomers)], Thiencarbazone-methyl, 6-Benzyladenine, Aminopyralid (sum of aminopyralid, its salts and its conjugates, expressed as aminopyralid), Amisulbrom, Flubendiamide, Meptyldinocap (sum of meptyldinocap and meptyldinocap phenol (2,4-DNMHP), expressed as meptyldinocap), Metaflumizone (sum of E- and Z-isomers), Imidacloprid, Methylisothiocyanate (resulting from the use of dazomet or metam), Hexythiazox (any ratio of constituent isomers), Oxyfluorfen, Pyroxsulam, Quinmerac (sum of quinmerac and its metabolites BH 518-2 and BH 518-4 expressed as quinmerac), Sulfuryl fluoride, Acequinocyl, Chlorantraniliprole, Emamectin B1a and its salts, expressed as emamectin B1a (free base), 1,4-dimethylnaphthalene, 8-hydroxyquinoline (sum of 8-hydroxyquinoline and its salts, expressed as 8-hydroxyquinoline), Sum of M4 and M6 (both free and conjugated), expressed as pinoxaden, Valifenalate, 1-methyl-3-(trifluoromethyl)-1*H*-pyrazole-4-carboxamide (PAM), Cycloxydim including degradation and reaction products which can be determined as 3-(3-thianyl) glutaric acid Sdioxide (BH 517-TGSO2) and/or 3-hydroxy-3-(3-thianyl)glutaric acid S-dioxide (BH 517-5-OHTGSO2) or derivatives thereof, calculated in total as cycloxydim, Cyflumetofen (sum of isomers), Penthiopyrad, Sum of metobromuron and 4-bromophenylurea, expressed as metobromuron, Isoxaben, Tetraconazole (sum of constituent isomers), Fipronil (sum fipronil (+) sulfone metabolite (MB46136) expressed as fipronil), Pyriproxyfen, Bicyclopyrone (sum of bicyclopyrone and its structurally related metabolites determined as the sum of the common moieties 2-(2-methoxyethoxymethyl)-6-(trifluoromethyl) pyridine-3-carboxylic acid (SYN503780) and (2-(2-hydroxyethoxymethyl)-6-(trifluoromethyl)pyridine-3-carboxylic acid (CSCD686480), expressed as bicyclopyrone), Triflumezopyrim, Chlormequat (sum of chlormequat and its salts, expressed as chlormequat-chloride), Chlorate, Oxadiargyl, Guazatine (guazatine acetate, sum of components), Atrazine, Tritosulfuron, Thiodicarb, Tricyclazole, Cinidon-ethyl, Triadimefon, Bitertanol (sum of isomers), Cyclaniliprole, Iprodione, Linuron, Fenbutatin oxide, Buprofezin, Diflubenzuron, Ethoxysulfuron, Ioxynil (sum of ioxynil and its salts, expressed as ioxynil), Molinate, Picoxystrobin, Amitrole, Flupyrsulfuronmethyl, Imazosulfuron, Isoproturon, Orthosulfamuron, Triasulfuron, Oxadiazon, Chlorothalonil, Ethoprophos, Fenamidone, Methiocarb (sum of methiocarb and methiocarb sulfoxide and sulfone, expressed as methiocarb), Propiconazole (sum of isomers), Dithiocarbamates (dithiocarbamates expressed as CS2, including maneb, mancozeb, metiram, propineb, thiram and ziram), Myclobutanyl, Pymetrozine, Propineb and its reaction products that are cleaved to propane-1,2-diamine (PDA), expressed as PDA, Propoxur, Thiram (expressed as thiram), Bromoxynil and its salts, expressed as bromoxynil, Chlorsulfuron, Epoxiconazole, Fenamiphos (sum of fenamiphos and its sulphoxide and sulphone expressed as fenamiphos), Triflumizole: Triflumizole and metabolite FM-6-1(*N*-(4-chloro-2-trifluoromethylphenyl)-*n*-propoxyacetamidine), expressed as Triflumizole, Phosmet, Novaluron (sum of constituent isomers), Bromopropylate, Chloridazon (sum of chloridazon and chloridazon-desphenyl, expressed as chloridazon), Imazaquin, Tralkoxydim (sum of the constituent isomers of tralkoxydim), Denatonium benzoate (sum of denatonium and its salts, expressed as denatonium benzoate), Etoxazole, Carbetamide (sum of carbetamide and its S isomer), Carboxin [carboxin plus its metabolites carboxin sulfoxide and oxycarboxin (carboxin sulfone), expressed as carboxin], Triflumuron, Oxamyl, Bispyribac (sum of bispyribac, its salts and its esters, expressed as bispyribac), Metosulam, Oryzalin, Oxasulfuron, Triazoxide	0.05 ^1^ mg/kg
Other apiculture products	Boscalid	0.15 mg/kg
Other apiculture products	Coumaphos Mercury compounds (sum of mercury compounds expressed as mercury)	0.01 mg/kg
Other apiculture products	Sodium 5-nitroguaiacolate, sodium o-nitrophenolate and sodium p-nitrophenolate (sum of sodium 5-nitroguaiacolate, sodium o-nitrophenolate and sodium p-nitrophenolate, expressed as sodium 5-nitroguaiacolate)	0.15 ^1^ mg/kg
Other apiculture products	Spirotetramat and spirotetramat-enol (sum of), expressed as spirotetramat	0.5 mg/kg
Other apiculture products	Ametoctradin	5 mg/kg
Other apiculture products	Fluoride ion	0.5 ^1^ mg/kg
Other apiculture products	2-amino-4-methoxy-6-(trifluormethyl)-1,3,5-triazine (AMTT), resulting from the use of tritosulfuron Tepraloxydim [sum of tepraloxydim and its metabolites that can be hydrolysed either to the moiety 3-(tetrahydropyran-4-yl)-glutaric acid or to the moiety 3-hydroxy-(tetrahydro-pyran-4-yl)-glutaric acid, expressed as tepraloxydim] Topramezone (BAS 670H)	0.1 ^1^ mg/kg

^1^ Limit of analytical determination.

## Data Availability

Not applicable.
